# Distance education project of English idioms learning from watching YouTube

**DOI:** 10.3389/fpsyg.2023.1171735

**Published:** 2023-07-03

**Authors:** Viphavee Vongpumivitch, Li-Tang Yu, Thuy Phuong Nguyen

**Affiliations:** ^1^Department of Foreign Languages and Literature, National Tsing Hua University, Hsinchu, Taiwan; ^2^Department of English Instruction, National Tsing Hua University, Hsinchu, Taiwan; ^3^Preparatory English Department, FPT University, Ho Chi Minh City, Vietnam

**Keywords:** English idioms, YouTube, note-taking, vocabulary learning, post-YouTube watching exercises

## Abstract

Idioms have been recognized to be commonly adopted by academic practitioners in their speech and writing. However, despite their common occurrence in English communication, idioms pose great challenges for non-native English speakers, as they are often ignored in English classrooms. Particularly in Vietnam, a minimal number of idioms appear in English textbooks published by the Ministry of Education, and teachers appeared to under-emphasize English idioms’ usefulness in conversation. To fill the gap, the present study adopted YouTube for Vietnamese students to undertake supplementary learning of English idiom knowledge. The study recruited 34 students and randomly assigned them to a control group and an experimental group. Both groups were required to take notes during their watching. Yet, only the experimental group would take extra exercises at the end of their watching. Immediate post-tests, delayed post-tests, and a survey were conducted. The findings demonstrate that the students in the two groups retained idiom knowledge similarly well. They were also positive towards learning English idioms through YouTube. Pedagogical implications and research suggestions are provided to conclude the study.

## 1. Introduction

Idioms, which are defined as an expression whose meaning is not “obvious from the individual words” (McCarthy and O’Dell, 2002, p.6), are frequently used in the daily life of native English speakers (Fernando, 1996). Idioms have also been recognized to be commonly adopted by academic practitioners in their speech and writing (Miller, 2020). Its importance for English users is undeniable. However, despite their common occurrence in English communication, idioms pose great challenges for non-native English speakers (Nippold, 1991), as they are often ignored in English classrooms (Cooper, 1998). Particularly in Vietnam where English is infrequently used outside English classrooms, few idioms appear in English textbooks published by the Ministry of Education, and teachers appeared to under-emphasize their usefulness in conversation (Tran, 2017). This disadvantages Vietnamese students’ learning outcomes of English idioms.

To equip students with idiomatic knowledge for daily conversation and academic communication, numerous instructional approaches have been posed (Boers, 2000; Zyzik, 2011; Eyckmans and Lindstromberg, 2016), and various studies have been conducted in conventional educational settings (Fotovatnia and Khaki, 2012; Abbasi et al., 2015). However, due to the widespread impact of COVID-19, schools were closed to minimize human contact and the spread of the virus. To keep learning in such circumstances, second-language learners engage in distance education and adopt online learning resources to foster their autonomous learning abilities (Gonzalez et al., 2020). Among rich learning materials, YouTube, one of the well-known, free, and popular video-sharing sites with a wealth of videos for English learning, has drawn the attention of second-language (L2) educators and researchers. However, little is known about the effect of watching YouTube videos on L2 learners’ idiom learning. Thus, the current study aims to explore how Vietnamese students increase their English idiom knowledge by watching English idiom-related videos on YouTube.

The following sections present reviews of relevant literature on using YouTube for second-language learning and supporting English-as-a-second-language learners to learn English idioms.

### 1.1. Literature review

#### 1.1.1. Using YouTube for second-language learning

The use of videos for learning is supported by cognitive science. As asserted by Mayer (2003), human beings can better manage information when receiving messages from dual channels: aural and visual, which handle auditory and visual input, respectively. This multimedia learning theory validates the use of YouTube for educational purposes.

In the L2 education field, YouTube is not foreign to teachers and students. Projects of multimodal video creation and sharing on YouTube have been initiated in L2 classes and demonstrated positive learning outcomes (Zhang et al., 2023). Moreover, learners can benefit from watching YouTube to enhance their L2 comprehension (Dizon, 2022). For example, Wang and Chen (2020) explored how EFL university students self-directed their English learning from YouTube. They realized that the students were highly positive about using YouTube in their English learning to find more learning materials, increase the motive of English learning, and gain more cultural awareness. Alobaid (2020) compared the L2 learners’ writing development before and after they incorporated YouTube into their writing tasks. The results showed that at the end of the research, the learners displayed a better writing performance. Kim and Kim (2021) found that watching YouTube videos successfully expanded the cultural horizon of Korean international college students studying in the U.S.A. Syafiq et al. (2021) and Saed et al. (2021) both confirmed that L2 learners could improve their speaking skills via YouTube videos as learning materials. Finally, Yaacob et al. (2021) demonstrated that young L2 English learners could boost their listening comprehension by watching YouTube videos and taking listening tasks.

YouTube also holds the potential for learning vocabulary and grammar. Specifically, YouTube videos can supplement in-class reading materials and help students boost vocabulary knowledge and reading comprehension (Kelsen, 2009). Watching YouTube videos containing newly learned vocabulary is helpful for short-term retention of vocabulary (Heriyanto, 2015). Furthermore, watching vlogs on YouTube leads to incidental learning of new vocabulary (Arndt and Woore, 2018). In addition to vocabulary learning, students with learning difficulties acquire grammar by watching videos that teach grammar (Huang, 2020).

In conclusion, YouTube benefits L2 learners by incorporating multimodal video creation in formal classes (Zhang et al., 2023). L2 learners watching YouTube videos also improve their skills (Dizon, 2022). More research is needed to enhance our understanding of the use of YouTube in more aspects of L2 learning, thus situating the research within the academic community and establishing its significance.

#### 1.1.2. Supporting English-as-a-second-language learners to learn English idioms via technology

Cucchiarini et al. (2022) asserted that information computer technology (ICT) with proper designs can create valid and effective contexts for learners to acquire L2 idioms. Numerous studies have investigated the utilization of technology in teaching and learning idioms. For example, mobile applications such as short message service (SMS) can facilitate idiom acquisition. Hayati et al. (2013) recruited 45 Persian participants from a private language institution and sorted them into three treatment groups (i.e., SMS, classroom learning, self-study) to learn 80 idioms. The SMS group received four messages daily. Each message was sent every three hours and contained one idiom, its definition, and a sample sentence. The students were only required to read the message without any instruction from teachers. The other groups studied idioms in conventional settings, either in regular language classes or in their self-study. The result showed that the SMS group outperformed the other two groups in their idiom recall tests. Moreover, Chen Hsieh et al. (2017) investigated both students’ retention and oral production of idioms by comparing the traditional in-classroom method and flipped classroom approach. The outcome favors the latter as English-as-a-foreign-language (EFL) participants gained higher scores in active recall tasks for idioms acquired using flipped classrooms, where learners received idiom information regularly via Line, an Internet-based instant messenger, and sent messages of their stories using learned idioms back to their peers and instructors.

In addition to sending SMS, an educational game called *Idiomatico* improves the participants’ receptive knowledge of English idioms. This result was found in a study conducted by Müller et al. (2017). The game developed by the research team presented learners with three possible meanings for each idiom. The score increased if the players got the correct answer and decreased if they got it wrong. Twenty-four Japanese learners and 14 Iranians joined the study. Japanese learners simply played it after class due to their tight school schedule, whereas the Iranian participants played the game with their friends and teacher during class time. Both groups significantly improved their idiom knowledge at the end of the research.

Videos have also been applied for idiom instruction. Sanaeifar (2017) found that students who learned idioms from both textbooks and animation videos recognized idioms better than those who learned only from textbooks. Wu et al. (2021) developed an animation/video-based application for Taiwanese EFL university students to learn English idioms. Positive findings were recorded based on learners’ test performance and interview responses. Moreover, Lin (2022) reported an intelligent system where YouTube videos were incorporated for supporting learners’ English-idiom learning. The system can evaluate speech rate and lexical difficulty of YouTube videos, design learning tasks like spelling games and fill-in-the-blanks, and recommend to learners YouTube videos appropriate to their learning progress. Yet, more empirical evidence is needed to demonstrate the efficacy of YouTube videos on L2 learners’ idiom learning.

During the pandemic, YouTube can offer beneficial learning resources for distance education. However, despite serving as a promising, rich learning-material repository, YouTube appeared not to be fully exploited for L2 learning. Learners simply pressed “like” and “share” buttons after watching videos without applying learning strategies, like note-taking, for deeper comprehension and retention (Wang and Chen, 2020). As research indicates that students perform differently even though they are exposed to the same learning materials, one of the possible explanations is how deeply they process information (Leow, 2018). The “[d]epth of processing is defined as the relative amount of cognitive effort, level of analysis, elaboration of intake together with the usage of prior knowledge, hypothesis testing, and rule formation employed in encoding and decoding some grammatical or lexical item in the input” (Leow and Mercer, 2015; p. 69). Thus, to support L2 learners’ further processing of the learning materials, the current study integrated an important learning strategy, note-taking, into L2 learners’ idiom learning during YouTube video watching, and examine whether the learners would have more solid idiom knowledge by taking extra exercises after video watching. The following research questions guide the study:

1. To what extent can learners learn idioms through watching YouTube videos? Can learners taking extra exercises outperform those without taking the exercises?

What are the students’ opinions about learning idioms through watching YouTube videos?

## 2. Methodology

The research adopted a quasi-experimental design to assign participants to a control group and an experimental group. Idiom tests and a survey were implemented to collect data for triangulation.

### 2.1. Participants

Thirty-four participants were recruited online from a Facebook group which consisted of Vietnamese students who were interested in pursuing higher-education degrees in Taiwan. All participants were native Vietnamese and familiar with information technology, particularly Internet surfing. They all voluntarily participated in the study and gave research consent to the researchers. None of the participants had lived or studied in an English-speaking country before participating in this study. Furthermore, their English proficiency was equal to or above the A2 Common European Framework of Reference for Languages (CEFR) level. To ensure that all participants’ English proficiency was statistically similar, all participants took the Cambridge English Test prior to watching the YouTube videos. The comparison of their test results showed no statistically significant difference (*p* > 0.97). Thus, both groups had similar English proficiency levels.

### 2.2. Youtube materials

Three YouTube channels were selected as the learning materials for the study, namely English with Max,^1^ English with Lucy,^2^ and AccurateEnglish.^3^ They were selected because of their high popularity among YouTube users with a large number of subscriptions. Moreover, the channels feature native speakers of English as teachers who teach idioms with similar styles to present English learning materials. For each idiom, the Youtubers read aloud its form and give oral explanations and examples of use. Concurrently, its written form, meaning, and example are demonstrated on the screen.

To select target idioms for the research, one of the researchers watched the YouTube videos and cross-checked the contents with *The Oxford Dictionary of Idioms* (Siefring, 2005) to confirm the expressions presented in those videos. Thirty-seven idioms from the videos on the three channels were selected. Then, a pilot vocabulary knowledge scale (VKS) test adapted from Wesche and Paribakht (1996) was conducted to examine if the participants recognized any of the 37 idioms. Finally, a list of 20 idioms was identified to be unknown to all participants. The idioms were equally distributed to 10 videos, each of which had two idioms.

### 2.3. Learning activity

The participants were first randomly assigned to either the control or experimental groups. To counterbalance the order of idioms that they learned, the participants in both groups were further divided into two sub-groups. Sub-group A in the control and experimental groups would watch videos 1–5 first and then videos 6–10, whereas sub-group B in the two groups would reverse the order.

Data were collected from one participant at a time. The third researcher held a weekly videoconferencing meeting with the control and experimental groups, respectively. The participants learned four or six idioms in each meeting. The third author served as the meeting moderator and shared her laptop screen with all participants to play every idiom video three times. In the second play, she would pause the videos so that the participants would take notes about the meaning, form, and example sentences of the idioms presented in the videos. They would send the photos of the hand-written notes via Facebook Messenger to the researchers immediately before they left the meeting. They were required to solely review their notes, but not watch the videos, to prepare for all the post-tests and delay post-tests.

It should be noted that the decision to require all participants to take notes while watching the YouTube videos did not simply come from our knowledge about active learning (*cf.* Wang and Chen, 2020). It was the participants’ request as well to “do something” while they watched the video so that they could feel that they were actively learning. This is the reason why in this study, both the control and experimental groups took notes, rather than having one group watching the videos without note-taking.

After sending the notes to the researchers, the experimental group would do idiom exercises to review what they had learned from the YouTube. They received Google Docs links to take the exercises. Each exercise had two tasks. The first exercise was a translation task. The meanings of the two newly learned idioms were provided in Vietnamese, and the experimental group gave their corresponding forms in English. The other task was a fill-in-the-blanks task. The experimental group wrote down the idioms that fitted the two short scenarios or dialogues provided in English. Once all participants in the experimental group submitted their exercises, the third researcher would post the answer key on the Facebook groups for the participants to check the results by themselves.

### 2.4. Research procedure

All participants had an immediate post-test once they finished the first 10 idioms. After they finished the other 10 idioms, they took the other immediate post-test. Finally, 3 weeks after the immediate post-tests, delayed post-tests were administered to investigate the retention of the target 20 English idioms. The research timeline is in Table 1.

**Table 1:** Research Timeline.

Week	Sub-group A task	Sub-group B task
1	English proficiency test; English idiom pre-test	
2	Videos 1, 2, and 3 (a total of six idioms)	Videos 4 and 5 (a total of four idioms)
3	Videos 4 and 5 (a total of four idioms); first post-test for the 10 idioms	Videos 1, 2, and 3 (a total of six idioms); first post-test for the 10 idioms
4	Videos 6, 7, and 8 (a total of six idioms)	Videos 9 and 10 (a total of four idioms)
5	Videos 9 and 10 (a total of four idioms); second post-test for the other 10 idioms	Videos 6, 7, and 8 (a total of six idioms); second post-test for the other 10 idioms
6	First delayed post-test for the first 10 idioms	
8	Second delayed post-test for the other 10 idioms	
9	Survey	

### 2.5. Instruments

Two instruments were used to collect data. Firstly, idiom tests were developed as an immediate post-test and a delayed post-test, aiming to examine the receptive and productive mastery of idiom knowledge (i.e., form and meaning). According to Nations (2014), when it comes to vocabulary learning, learners must be able to recall the meanings when they are presented with the target words (i.e., meaning and form). Learners have achieved productive mastery of vocabulary meaning when they can recall the target words when related contexts or ideas are given (i.e., meaning association) and produce the correct forms of the target words when meanings are given (i.e., meaning and form). Furthermore, learners need to know how to write and spell the target words to achieve productive mastery of vocabulary’s form (i.e., written form). Therefore, four parts were designed in the post-tests to measure these aspects of idiomatic knowledge.

The first task aimed at measuring receptive knowledge of form and meaning. It required the participants to match the given idioms with their corresponding definitions (i.e., idioms and definition matching). The definitions were written in English. The second task aimed at measuring productive knowledge of form and meaning. The participants chose the appropriate idioms in the given contexts (i.e., multiple choices). The third task aimed at measuring productive knowledge of idioms’ forms. It required the participants to correct mistakes found in idioms’ forms (i.e., error correction). The errors were created by replacing the correct constituent parts with synonyms or words belonging to the same topic group. Finally, the fourth task aimed at measuring productive knowledge of form and meaning, asking the participants to translate the bolded parts written in Vietnamese into English, and the translated parts must include the target idioms. Each item was worth one point. If the participants answered the item correctly, they would be awarded one point. No partial score was given.

To ensure the validity and reliability of the designed tests, all test items were examined by specialists in the field of L2 education and revised based on their suggestions. Once the test items were confirmed, they were produced on Google Docs. The third researcher sent to a group of English learners the link of the Google Docs file to undertake a pilot test, thus streamlining test administration. The test items in the immediate post-test and the delayed post-test were the same but in a different order.

The other instrument was a survey to explore the participants’ attitudes towards watching YouTube videos to learn English idioms. There were four questions. The first question asked about the participants’ enjoyment of learning idioms by watching YouTube with a five-point Likert scale from extremely dislike (1) to extremely like (5). The following multiple-choice question investigated participants’ plans to continue using YouTube to learn idioms with four options. Finally, two open-ended questions were posed to investigate how the participants liked learning idioms on YouTube and the factors that affected their idiom learning via watching videos on YouTube. The survey items were written in both Vietnamese and English. The participants responded in their preferred language. The survey items were also reviewed by specialists in the field of L2 education.

### 2.6. Data collection and analysis

Two types of data were collected: idiom test outcomes and survey responses. The test outcomes comprised receptive knowledge learning performance (Task 1) and productive knowledge learning performance (Tasks 2–4). The scores of Tasks 2–4 were averaged. Due to a small sample size, non-parametric statistical approaches were conducted. Mann–Whitney U Tests were used to examine the difference between the two groups, and Wilcoxon Signed Rank Tests were conducted to examine the difference between the immediate post-tests and the delayed post-tests.

Regarding survey responses, descriptive statistics were adopted to calculate the mean and the standard deviation of the Likert-scale item and the frequency of selected options of the multiple choices. Finally, content analysis (Patton, 2015) was conducted to examine the responses to the open-ended questions. Similar ideas were organized into categories by the researchers. Disagreements on categorization were resolved through discussion.

## 3. Results

Q1. To what extent can learners learn idioms through watching YouTube videos? Can learners taking extra exercises outperform those without taking the exercises?

Table 2 shows the means and standard deviations of the immediate post-tests and the delayed post-tests outcome. The averages of the performance in receptive and productive knowledge of all tests were at least more than eight out of 10, the full score. To examine whether taking additional idiom exercises after video-watching benefited participants’ learning, Mann–Whitney U tests were administered to analyze all test outcomes of the control and experimental groups in Sub-group A and Sub-group B. No significant difference was found between the control and experimental groups’ Sub-group A and Sub-group B (*p* > 0.05), suggesting that merely watching videos and taking notes could result in learning improvement.

**Table 2:** Descriptive statistics result of the idiom tests and the result of Mann–Whitney *U* Tests.

	Sub-group A			Sub-group B		
Group test	Control group (*n* = 8)	Experimental group (*n* = 9)	Z^b^ (value of *p*)	Control group (*n* = 9)	Experimental group (*n* = 8)	Z^b^ (value of *p*)
1st immediate post-test: receptive knowledge	8.50 (2.83)^a^	9.33 (1.00)	0.00 (1.00)	8.44 (2.46)	8.50 (3.21)	−0.30 (0.77)
1st immediate post-test: productive knowledge	9.29 (1.31)	9.48 (0.88)	0.00 (1.00)	8.85 (1.72)	8.83 (2.33)	−0.27 (0.79)
2nd immediate post-test: receptive knowledge	9.13 (1.81)	8.56 (2.51)	−0.42 (0.68)	8.22 (2.59)	9.50 (0.93)	−1.02 (0.31)
2nd immediate post-test: productive knowledge	9.21 (1.38)	9.22 (1.76)	−0.17 (0.87)	8.81 (1.84)	8.92 (1.88)	−0.32 (0.75)
1st delayed post-test: receptive knowledge	9.13 (1.64)	9.22 (2.33)	−0.58 (0.56)	8.56 (3.36)	8.75 (2.82)	0.00 (1.00)
1st delayed post-test: productive knowledge	9.50 (0.94)	9.67 (0.58)	−0.06 (0.95)	9.41 (0.76)	8.83 (1.77)	−0.32 (0.75)
2nd delayed post-test: receptive knowledge	9.50 (0.93)	8.67 (3.04)	−0.13 (0.90)	8.89 (2.42)	9.25 (2.12)	−0.51 (0.61)
2nd delayed post-test: productive knowledge	9.13 (1.46)	9.37 (1.55)	−0.60 (0.55)	8.78 (1.67)	8.67 (2.34)	−0.22 (0.83)

^a^ Mean (standard deviation).

^b^ Mann–Whitney *U* Test.

Wilcoxon Signed Ranks Test was implemented to examine whether the participants in the two groups could retain their idiom knowledge 3 weeks after their learning. A comparison between the immediate post-tests and the delayed post-tests of the control and experimental groups was undertaken. As Table 3 displays, non-significant findings between the two tests were detected. Thus, it seems that the participants in the two groups could perform similarly in the immediate post-tests and the delayed post-tests. In other words, the effect of watching YouTube videos and taking notes to learn English idioms on the participants’ learning could be maintained for 3 weeks.

**Table 3:** Wilcoxon signed ranks test result of immediate post-test and delayed post-test comparison.

	Sub-group A		Sub-group B	
Group	Control group (*n* = 8)	Experimental group (*n* = 9)	Control group (*n* = 9)	Experimental group (*n* = 8)
Test				
1st immediate post-test-Receptive knowledge-1st delayed post-test: receptive knowledge	8.50 vs. 9.13^a^	9.33 vs. 9.22	8.44 vs. 8.56	8.50 vs. 8.75
	−1.34 (0.18)^b^	0.00 (1.00)	0.00 (1.00)	−0.54 (0.59)
1st immediate post-test: productive knowledge- 1st delayed post-test: productive knowledge	9.29 vs. 9.50	9.48 vs. 9.67	8.85 vs. 9.41	8.83 vs. 8.83
	−1.34 (0.18)	−1.34 (0.18)	−1.84 (0.07)	0.00 (1.00)
2nd immediate post-test: receptive knowledge- 2nd delayed post-test-Receptive knowledge	9.13 vs. 9.50	8.56 vs. 8.67	8.22 vs. 8.89	9.50 vs. 9.25
	−0.45 (0.66)	0.00 (1.00)	−0.55 (0.58)	−0.45 (0.66)
2nd immediate post-test: productive knowledge- 2nd delayed post-test-Productive knowledge	9.21 vs. 9.13	9.22 vs. 9.37	8.81 vs. 8.76	8.92 vs. 8.67
	−0.58 (0.56)	−0.54 (0.59)	−0.37 (0.71)	−1.07 (0.29)

^a^ Within-group’s two means.

^b^ Z (*p* value).

Q2. What are the students’ opinions towards learning idioms by watching YouTube videos?

A survey was launched at the end of data collection to obtain all participants’ opinions towards learning idioms by watching YouTube videos. Table 4 demonstrates the result of the Likert-scale and multiple-choice items. Both groups displayed positive attitudes towards watching YouTube to learn English idioms. The majority of the participants either liked or extremely liked the learning approach. When asked if they would continue using YouTube for idiom learning, all participants were willing to do so. Over half of the participants would further search for more learning materials on YouTube in the future.

**Table 4:** The result of the Likert-scale and multiple-choice items.

	Okay	Like	Extremely like	Mean	Standard deviation	Median
Enjoyment of Watching YouTube videos to learn English idioms						
Control Group (*n* = 17)	3 (18%)	12 (71%)	2 (12%)	3.94	0.56	4.00
Experimental Group (*n* = 17)	4 (24%)	10 (59%)	3 (18%)	3.94	0.66	4.00
Plan to use YouTube for English idiom learning in the future						
	No, I will not use YouTube to learn idioms. There are other better ways to learn.	Yes, I will continue to use YouTube to learn idioms, but I will find new YouTubers on my own.		Yes, I will continue to use YouTube to learn idioms. I will continue with these same YouTubers in this experiment.	Yes, I will continue to use YouTube to learn idioms. I will both continue with these same YouTubers in this experiment and find new YouTubers on my own.	
Control group (*n* = 17)	0	4 (24%)		4 (24%)	9 (53%)	
Experimental group (*n* = 17)	0	0		8 (47%)	9 (53%)	

The participants were asked in the survey to offer their viewpoints about watching YouTube to learn English idioms. They positively viewed YouTube videos. The top three advantages were *clear pronunciation and beautiful voice*, *attractive teaching style*, and *the short duration of the videos*. When reflecting on their learning outcome from watching YouTube videos, the participants used a variety of tips, including *reviewing the idioms before the quizzes*, *staying confident, calm, and ready*, and *applying learning strategies, like using learned idioms in daily life and deducing idioms’ meanings from their keywords*. Thus, they made progress in their idiom learning.

## 4. Discussion and conclusion

Our research findings demonstrated that YouTube was indeed a valuable source of learning idioms and note-taking was a helpful strategy. Over 60 percent of the participants in both groups achieved the perfect 10 score in the immediate post-tests and the delayed post-tests. Their performance indicated that they gained considerable idiom knowledge by watching YouTube and taking notes. This is consistent with previous studies which found that YouTube was beneficial in learning different aspects of the English language, like vocabulary and grammar (Heriyanto, 2015; Arndt and Woore, 2018; Huang, 2020). In line with Wang and Chen’s (2020) study, the participants also displayed positive attitudes towards watching YouTube for English-learning purposes. The current study, therefore, corroborates the argument that learners should take advantage of YouTube to learn L2, especially idioms that are often under-emphasized in traditional language classes.

Moreover, our findings strengthen the belief that ICT tools are full of potential for learning and teaching English idioms (Cucchiarini et al., 2022). Complement with previous studies using SMS (Hayati et al., 2013), Line App (Chen Hsieh et al., 2017), educational computer games (Müller et al., 2017), and animation video (Sanaeifar, 2017) for idiom learning, YouTube is an accessible source of learning materials for L2 learners to receive comprehensible input. In addition, note-taking offered an appropriate amount of information processing (Leow and Mercer, 2015; Leow, 2018), enabling the participants to retain receptive and productive knowledge of idioms.

The study implies that watching YouTube videos could be one of the effective ways to learn L2. For those who want to self-study and have access to the Internet, YouTube can be a convenient, affordable, and accessible venue. Next, since the participants remembered well the target idioms by watching YouTube and taking notes without extra exercises, learners who wish to study idioms independently can apply this note-taking strategy in their self-directed learning *via* YouTube to achieve a suitable level of information processing. Thus, language teachers can instruct students in note-taking skills and encourage or guide students to learn idioms by selecting and presenting suitable YouTube videos.

The current study’s participants’ very high motivation for English learning could affect the positive outcomes. Additionally, several limitations affect the generalizability of the research findings, such as the small sample size, the short duration of research, the basic proficiency levels of participants, the limited number of idioms tested and qualitative data collected, and no control group who watched the YouTube videos without taking notes and doing exercises. These issues need to be addressed in future studies. Finally, future studies can adopt a variety of ways to observe how L2 learners watch YouTube videos and take notes and collect relevant data through interviews and observations. It is of interest to explore how many idioms per YouTube videos are manageable for L2 learners.

## Data availability statement

The original contributions presented in the study are included in the article/supplementary material, further inquiries can be directed to the corresponding author/s.

## Ethics statement

Ethical review and approval was not required for the study on human participants in accordance with the local legislation and institutional requirements. Written informed consent to participate in this study was provided by the participants’ legal guardian/next of kin.

## Author contributions

VV and TPN: conceptualization, methodology, and project administration. VV, L-TY, and TPN: writing—original draft and formal analysis. VV and L-TY: writing—review and editing. All authors contributed to the article and approved the submitted version.

## Funding

The Article Processing Charge is funded by the National Science and Technology Council, Taiwan, under grant numbers 111-2410-H-007-036.

## Conflict of interest

The authors declare that the research was conducted in the absence of any commercial or financial relationships that could be construed as a potential conflict of interest.

## Publisher’s note

All claims expressed in this article are solely those of the authors and do not necessarily represent those of their affiliated organizations, or those of the publisher, the editors and the reviewers. Any product that may be evaluated in this article, or claim that may be made by its manufacturer, is not guaranteed or endorsed by the publisher.

## Footnotes

^1^ https://www.youtube.com/@EnglishwithMax

^2^ https://www.youtube.com/@EnglishwithLucy

^3^ https://www.youtube.com/@AccurateEnglish
